# Analysis of risk factors and construction of nomograph model for critical condition of patients with hypertension during pregnancy

**DOI:** 10.1186/s12884-023-05860-7

**Published:** 2023-08-10

**Authors:** Pingping Zhang, Xiwen Zhong

**Affiliations:** Department of Gynaecology and Obstetrics, Wenzhou Central Hospital, No.252, Baili East Road, Lucheng District, Wenzhou City, 325000 Zhejiang Province China

**Keywords:** Hypertension during pregnancy, Critical, Risk factors, Nomogram

## Abstract

**Objective:**

This study aims to construct the risk prediction nomogram model of critical condition in patients with hypertension during pregnancy and to verify its evaluation effect.

**Methods:**

A total of 531 patients with hypertension during pregnancy were randomly grouped into 427 model group and 104 validation group. The model group patients included 59 cases of critical group and 368 cases of non-critical group according to the occurrence of critical situation. Multivariate Logistic regression analysis was conducted to determine the risk factors of critical condition in patients with hypertension during pregnancy, and R software was used to construct the nomogram model. Moreover, the prediction efficiency of the model was evaluated.

**Results:**

The proportions of patients aged over 30 years, with an educational background of junior high school or below, a family history of hypertension, anemia during pregnancy, and a lower erythrocyte count were significantly higher in the critical group compared to the non-critical group (P < 0.05). Age > 30 years old, educational background of junior high school and below, family history of hypertension, anemia during pregnancy, and red blood cell count were independent risk factors for the occurrence of critical condition in patients with hypertension during pregnancy (P < 0.05). The prediction model formula Z = 1.857×Age + 1.167×Education + 1.601×Family history of hypertension + 1.815×Pregnancy anemia + 3.524×Red blood cell count+(-19.769). The area under the curve (AUC) of the nomogram in the modeling group for predicting the risk of critical situations was 0.926 (95% CI = 0.887 ~ 0.964), indicating excellent discrimination. The calibration curve closely resembled the ideal curve, demonstrating good agreement between the predicted and actual values. The AUC of the validation group’s nomogram to predict the risk of critical situation was 0.942 (95% CI = 0.872 ~ 0.998), with good discrimination. The calibration curve was close to the ideal curve, and the actual value was in good agreement with the predicted value.

**Conclusion:**

The nomograph model can predict the risk of critical condition in patients with hypertension during pregnancy and screen high-risk population.

## Introduction

Hypertension in pregnancy, including gestational hypertension, preeclampsia, and eclampsia, is a pervasive global health issue affecting approximately 10% of pregnant women worldwide [1]. Hypertension in pregnancy not only involves the heart, brain, and other systemic organs of pregnant women, but also has close association with pregnancy complications and increased neonatal mortality, seriously threatening the lives of pregnant women and newborns [2]. While the number of maternal deaths related to hypertension has decreased both domestically and internationally in recent years, hypertension in pregnancy remains a pressing issue due to its significant impact on maternal and perinatal outcomes [3]. Individuals at higher risk of severe preeclampsia may require more frequent monitoring due to risk factors such as age, high BMI, or existing medical conditions [4]. To date, numerous clinical studies have investigated the factors influencing the development of hypertension in pregnancy. However, the factors that contribute to the development of critical conditions in patients with hypertension during pregnancy remain poorly defined [5, 6]. In order to identify the risk factors for the development of critical conditions in patients with hypertension in pregnancy and to improve the prevention and treatment of critical patients, 531 cases of patients with hypertension in pregnancy admitted to our hospital were selected in this study to investigate the risk factors for the development of critical conditions. A risk prediction nomogram model was constructed to provide reference for early screening of high-risk groups and development of reasonable interventions in the future.

## Data and methods

### General data

**Fig. 1:**
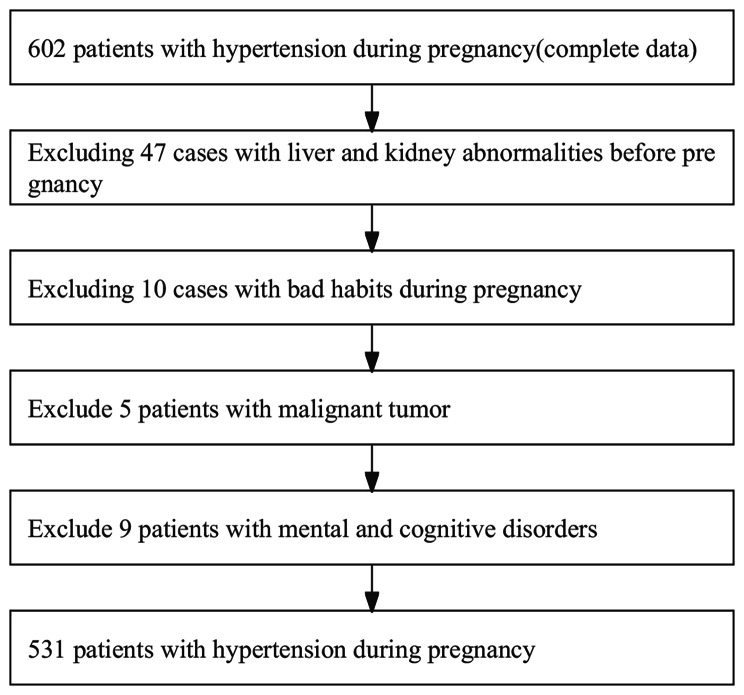
Flow chart of case collection

The 531 gestational hypertension patients admitted to our hospital between October 2019 and January 2023 were randomly divided into a modeling group (427 cases) and a validation group (104 cases). Within the modeling group, patients were further categorized into a critical group (59 cases) and a non-critical group (368 cases) based on the presence or absence of critical conditions. Severe preeclampsia was classified as critical, while gestational hypertension and mild preeclampsia were classified as non-critical. The study was approved by the clinical ethics committee of our hospital and met the ethical standards, with informed consent obtained from the patients and their families. As shown in Fig. 1.

Inclusion criteria were as follows: ① patients diagnosed with hypertension in pregnancy according to international diagnostic criteria [7]; ② good compliance, with patients and family members able to actively participate in the study and treatment, ensuring complete recording of all data; ③ singleton pregnancies. Exclusion criteria were: ① pre-existing liver and kidney abnormalities before pregnancy; ② engagement in harmful habits during pregnancy; ③ presence of concomitant malignancies; ④ presence of mental or cognitive impairments.

### Data collection

Clinical data of patients with hypertension in pregnancy were collected and recorded, including age (≤ 30 years, > 30 years), BMI (≤ 28 kg/m^2^, > 28 kg/m^2^), education (high school and above, junior high school and below), living environment (urban, rural), family history of hypertension (none, yes), week of pregnancy (≤ 30 weeks, > 30 weeks), number of pregnancies (1, ≥ 2), number of deliveries (< 2, ≥ 2 times), anemia during pregnancy (none, yes), gestational nephropathy (none, yes), gestational diabetes (none, yes), platelet count, and red blood cell count.

### Statistical processing

SPSS 25.0 statistical software was used for analysis. The measurement data conformed to normal distribution by ($\bar x\, \pm \,s$), and *t* test was performed between the groups; the count data were expressed as n (%), and ${\chi ^2}$ test was performed between the groups; multiple Logistic regression analysis was performed to determine the risk factors for the development of critical conditions in gestational hypertension patients. R software (R3.5) and rms program package were used to construct a nomogram model for predicting the risk of critical conditions in gestational hypertension patients. Receiver operating curve (ROC) was plotted, and the area under curve (AUC) was calculated to evaluate the predictive value and discrimination of the nomogram model. *P* < 0.05 indicates statistically significant difference.

## Results

### Comparison of clinical data between the modeling group and the validation group

There was no statistically significant difference between the modeling group and the validation group in terms of age, BMI, education, living environment, family history of hypertension, gestational week, number of pregnancies, number of deliveries, anemia during pregnancy, gestational nephropathy, gestational diabetes, platelet count, and red blood cell count (*P* > 0.05), as shown in Table 1.

**Table 1:** Comparison of clinical information between the modeling group and the validation group

index	Modeling group(n = 427)	Validation group(n = 104)	${\chi ^2}/t$	*P*
Age(years old)			0.101	0.751
≤ 30	225(52.69)	53(50.96)		
>30	202(47.31)	51(49.04)		
BMI(kg/m^2^)			0.118	0.731
≤ 28	309(72.37)	77(74.04)		
>28	118(27.63)	27(25.96)		
education[n(%)]			0.296	0.587
High school and above	242(56.67)	62(59.62)		
Junior high school and below	185(43.33)	42(40.38)		
Residential environment[n(%)]			2.367	0.124
city	269(63.00)	57(54.81)		
countryside	158(37.00)	47(45.19)		
history of hypertension[n(%)]			2.193	0.139
No	315(73.77)	84(80.77)		
Yes	112(26.23)	20(19.23)		
Gestational week(week)			0.085	0.770
≤ 30	208(48.71)	49(47.12)		
>30	219(51.29)	55(52.88)		
Number of pregnancies(number)			0.000	0.997
1	230(53.86)	56(53.85)		
≥ 2	197(46.14)	48(46.15)		
Number of births(number)			0.078	0.780
<2	244(57.14)	61(58.65)		
≥ 2	183(42.86)	43(41.35)		
Anemia during pregnancy[n(%)]			3.288	0.070
No	294(68.85)	81(77.88)		
Yes	133(31.15)	23(22.12)		
Nephrosis complicating pregnancy[n(%)]			1.761	0.184
No	334(78.22)	75(72.12)		
Yes	93(21.78)	29(27.88)		
Gestational diabetes[n(%)]			0.002	0.969
No	303(70.96)	74(71.15)		
Yes	124(29.04)	30(28.85)		
platelet count(×10^9^/L)	178.94 ± 60.96	180.25 ± 66.31	0.193	0.847
Red blood cell count(×10^12^/L)	3.97 ± 0.59	3.96 ± 0.62	0.153	0.878

### Comparison of clinical data between critical and non-critical patients in the modeling group

There was no statistically significant difference between the non-critical group and the critical group in terms of BMI, living environment, gestational week, number of pregnancies, number of deliveries, gestational nephropathy, gestational diabetes, platelet count, and red blood cell count (*P* > 0.05). The proportion of patients with age > 30 years old, education of junior high school or below, family history of hypertension, anemia during pregnancy and red blood cell count were higher in the critical group than in the non-critical group, showing statistically significant differences (*P* < 0.05), as shown in Table 2.

**Table 2:** Comparison of clinical data between critical and non-critical patients in the modeling group

Index	No critical group(n = 368)	Critical group(n = 59)	${\chi ^2}/t$	*P*
Age(years old)			28.749	0.000
≤ 30	213(57.88)	12(20.34)		
>30	155(42.12)	47(79.66)		
BMI(kg/m^2^)			0.715	0.398
≤ 28	269(73.10)	40(67.80)		
>28	99(26.90)	19(32.20)		
Education[n(%)]			14.463	0.000
High school and above	222(60.33)	20(33.90)		
Junior high school and below	146(39.67)	39(66.10)		
Residential environment[n(%)]			0.058	0.809
city	231(62.77)	38(64.41)		
countryside	137(37.23)	21(35.59)		
history of hypertension[n(%)]			34.878	0.000
No	290(78.80)	25(42.37)		
Yes	78(21.20)	34(57.63)		
Gestational week(week)			0.238	0.625
≤ 30	181(49.18)	27(45.76)		
>30	187(50.82)	32(54.24)		
Number of pregnancies(number)			1.409	0.235
1	194(52.72)	36(61.02)		
≥ 2	174(47.28)	23(38.98)		
Number of births(number)			2.244	0.134
<2	205(55.71)	39(66.10)		
≥ 2	163(44.29)	20(33.90)		
Anemia during pregnancy[n(%)]			35.311	0.000
No	273(74.18)	21(35.59)		
Yes	95(25.82)	38(64.41)		
Nephrosis complicating pregnancy[n(%)]			0.938	0.333
No	285(77.45)	49(83.05)		
Yes	83(22.55)	10(16.95)		
Gestational diabetes[n(%)]			0.002	0.967
No	261(70.92)	42(71.19)		
Yes	107(29.08)	17(28.81)		
Platelet count(×10^9^/L)	177.94 ± 62.97	185.02 ± 66.91	0.795	0.427
Red blood cell count(×10^12^/L)	3.90 ± 0.42	4.36 ± 0.48	7.652	0.000

### Multiple logistic regression analysis of the development of critical conditions in patients with hypertension in pregnancy

The occurrence of critical conditions in patients with hypertension during pregnancy was used as the dependent variable, with a value of 1 indicating presence and 0 indicating absence. Age (1 = > 30 years, 0 = ≤ 30 years), education (1 = junior high school and below, 0 = high school and above), family history of hypertension (1 = yes, 0 = no), anemia during pregnancy (1 = yes, 0 = no), and red blood cell count (as a continuous variable) were selected as independent variables for multiple logistic regression analysis. These variables were chosen based on their statistically significant differences observed in the aforementioned univariate analysis. The results showed that age > 30 years, education of junior high school and below, family history of hypertension, presence of anemia during pregnancy, and red blood cell count were independent risk factors for the development of critical conditions in patients with hypertension in pregnancy (*P* < 0.05). The prediction model formula is Z = 1.857×Age + 1.167×Education + 1.601×Family history of hypertension + 1.815×Pregnancy anemia + 3.524×Red blood cell count+(-19.769). as shown in Table 3.

**Table 3:** Multiple Logistic regression analysis of the development of critical conditions in patients with hypertension in pregnancy

influence factor	B	SE	Wald	OR	95%CI	*P*
Age	1.857	0.452	16.909	6.407	2.644 ~ 15.530	0.000
Education	1.167	0.404	8.347	3.212	1.455 ~ 7.090	0.004
history of hypertension	1.601	0.405	15.657	4.959	2.244 ~ 10.961	0.000
Anemia during pregnancy	1.815	0.408	19.775	6.139	2.759 ~ 13.661	0.000
Red blood cell count	3.524	0.536	43.235	33.908	11.862 ~ 96.927	0.000
constant	-19.769	2.510	62.041	0.000	-	0.000

### Construction of a risk prediction nomogram model for the multiple Logistic regression analysis on the development of critical illness in patients with hypertension in pregnancy

**Fig. 2:**
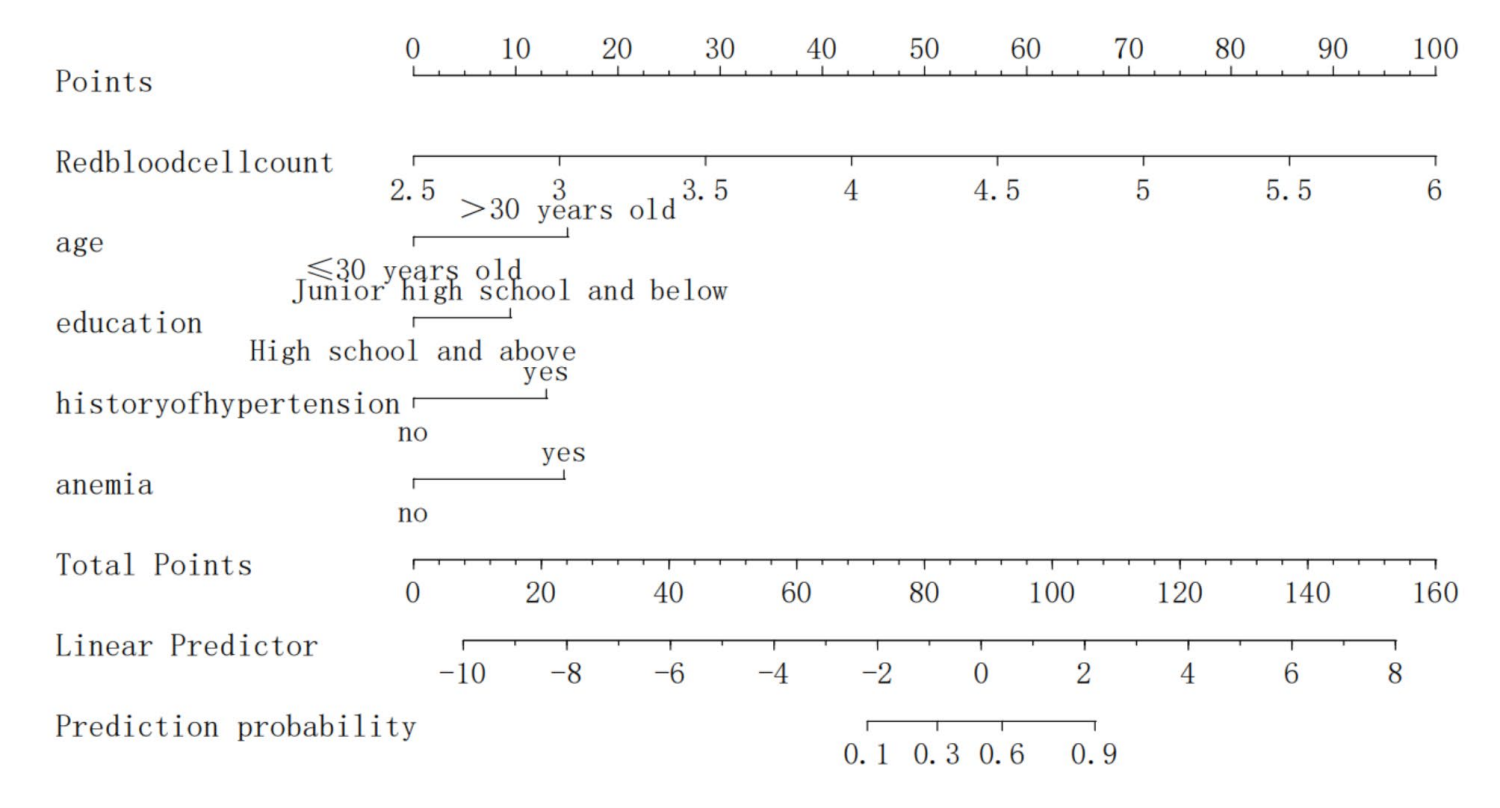
Nomogram model for predicting the risk of critical conditions in patients with hypertension in pregnancy

Based on the results of the multiple logistic regression analysis described above, a nomogram model was developed to predict the risk of critical conditions in patients with hypertension during pregnancy. The model assigns 14.9 points for age > 30 years old, 8.6 points for junior high school education or below, 12.5 points for a family history of hypertension, 13.7 points for anemia during pregnancy, and 14.6 points for every 0.5 × 1012/L increase in red blood cell count. The visualization of this model is presented in Fig. 2.

### Internal validation of the nomogram model for predicting the risk of critical conditions in patients with hypertension in pregnancy

**Fig. 3:**
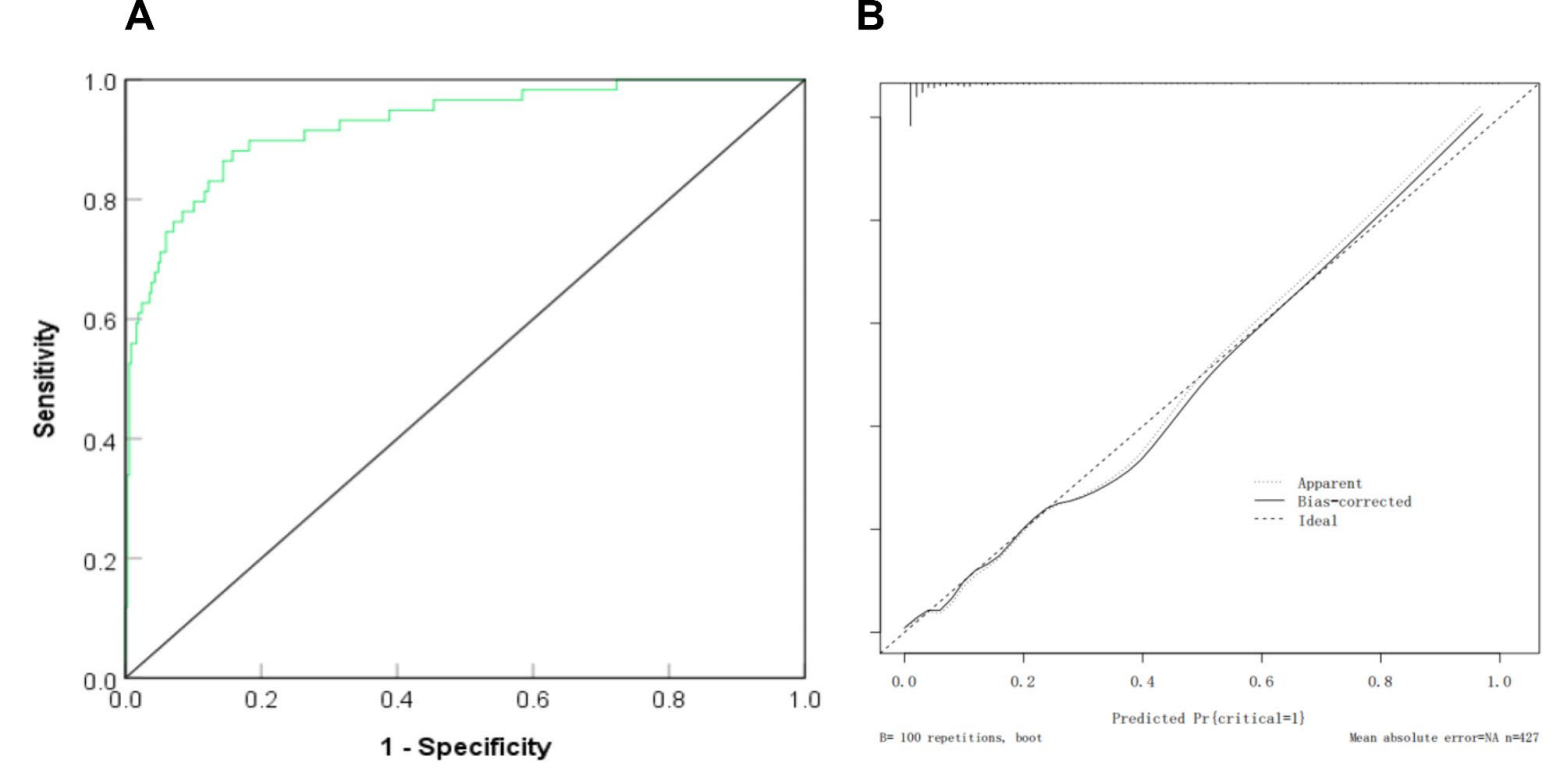
Receiver operating characteristic and correction curve of nomogram model for predicting the severity of hypertensive patients during pregnancy

For the modeling group, ROC curve was plotted to validate discrimination of the nomogram prediction model. The results showed that the AUC of the nomogram for predicting the risk of critical conditions was 0.926 (95% CI = 0.887–0.964), with good discrimination, as shown in Fig. 3A. The calibration curve tended to be close to the ideal curve, and the actual values agreed well with the predicted values, as shown in Fig. 3B.

### External validation of the nomogram model for predicting the risk of critical conditions in patients with hypertension in pregnancy

**Fig. 4:**
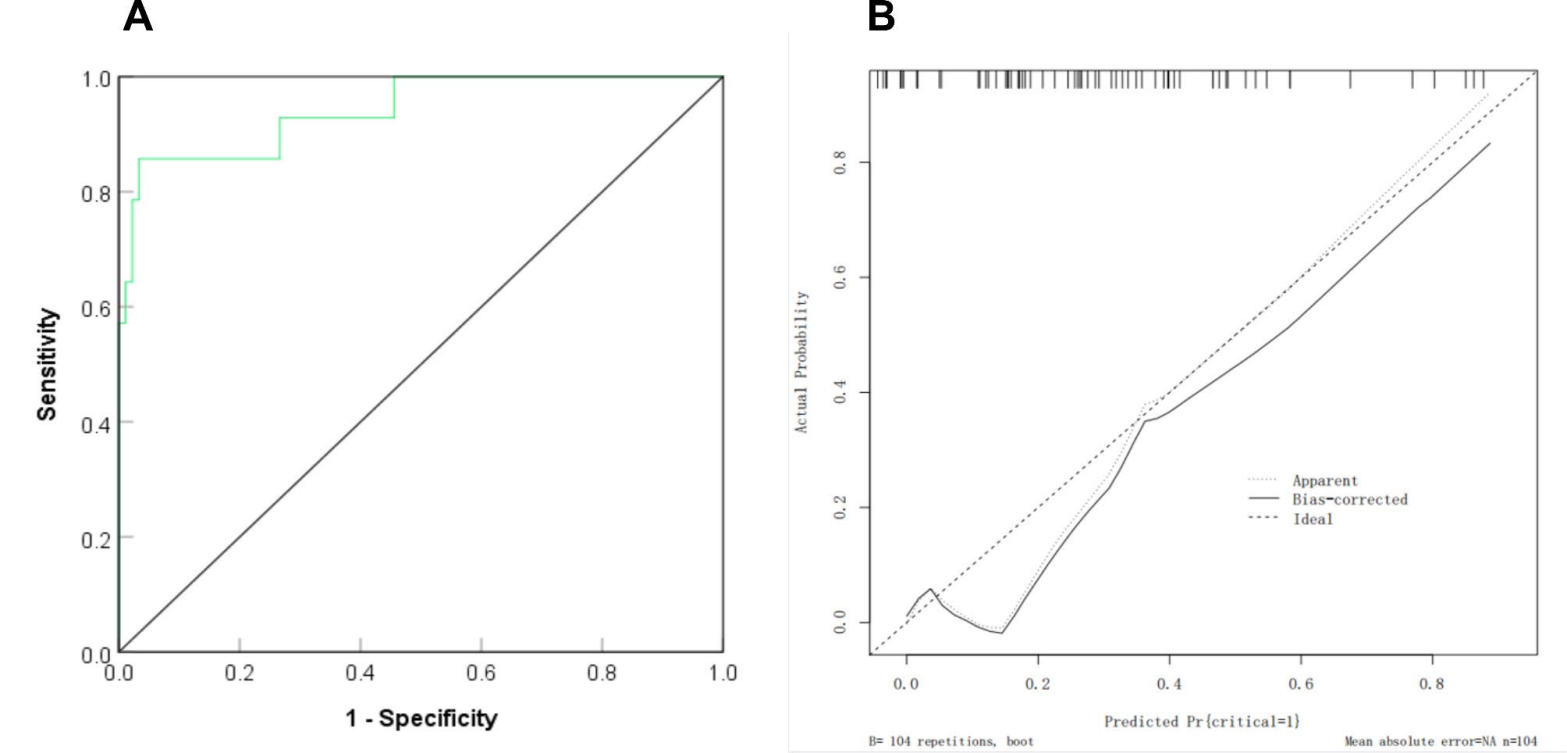
Receiver operating characteristic and correction curve of nomogram model for predicting the severity of hypertensive patients during pregnancy

In the validation group, the AUC of the nomogram for predicting the risk of critical condition was 0.942 (95% CI = 0.872–0.998), with good discrimination, as shown in Fig. 4A. The calibration curve tended to be close to the ideal curve, and the actual values agreed well with the predicted values, as shown in Fig. 4B.

### Importance score of risk factors for critical situations in patients with gestational hypertension

**Fig. 5:**
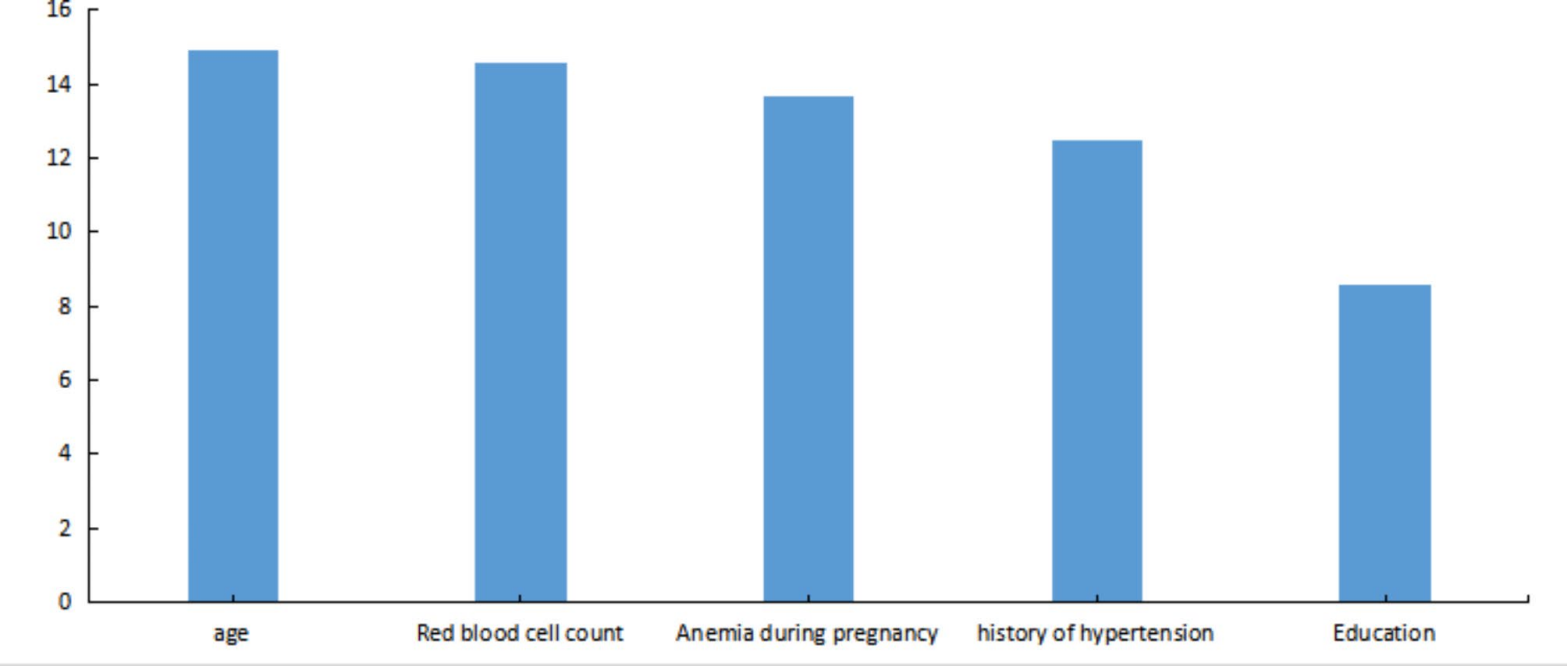
Importance score of prediction variables of Random forest model

Based on the Gini coefficient, which measured the importance score of each variable, the results indicated that age was the most influential factor impacting critical conditions in hypertensive patients during pregnancy. Following age, the variables in descending order of importance were red blood cell count, anemia during pregnancy, family history of hypertension, and educational background, as shown in Fig. 5.

## Discussion

As one of the important obstetric diseases, severe preeclampsia is a major cause of maternal and infant morbidity and mortality [8]. Clinical manifestations of preeclampsia include headache, dizziness, nausea, stomach pain, and vomiting, and its common short-term complications include fetal growth restriction, iatrogenic proterm labor, and chronic intrauterine hypoxia [9, 10]. In the long term, individuals who have experienced preeclampsia face an elevated risk of developing heart disease, cerebrovascular disease, diabetes, and cognitive impairment [11]. Pre-eclampsia is often underestimated in clinical practice, and some individuals with severe pre-eclampsia may initially present with mild symptoms. However, the condition can progress rapidly, and when patients develop neurological complications such as cerebral edema and cerebral hemorrhage, the mortality rate can reach as high as 70%. Therefore, it is crucial to remain vigilant and promptly identify and manage pre-eclampsia to mitigate the risk of severe complications [12, 13]. Therefore, construction of individualized model for predicting the risk of critical conditions in patients with hypertension in pregnancy carries important significance for guiding treatment and improving prognosis.

The results of multiple Logistic regression analysis in this study showed that age > 30 years, education of junior high school or below, family history of hypertension, presence of anemia during pregnancy, and red blood cell count were independent risk factors affecting the development of critical conditions in patients with hypertension in pregnancy. (1) Age. As puerpera age increases, their uterus and other organ functions may tend to decrease or even decline, and older puerpera has significantly lower pregnancy tolerance than younger puerpera, so their vascular resistance, pregnancy complications, etc. will increase accordingly, with the risk of critical conditions increased [14, 15]. (2) Education. Less educated patients with hypertension in pregnancy have relatively weak health care awareness, who rarely receive perinatal care and prenatal checkups on schedule. As a result, development of hypertension in pregnancy from mild to severe conditions cannot be identified early. (3) Family history of hypertension. Hypertension in pregnancy may have a certain genetic predisposition, and pregnant women with a family history of hypertension may have an increased risk of developing hypertension in pregnancy and aggravating the condition [16]. (4) Anemia during pregnancy. Anemia during pregnancy can trigger a decrease in the body immune function of the puerpera. The inability of the blood supply to meet the fetus needs, and the lack of placental nutrients and other perfusion capacity, plus the high blood pressure in patients with gestational hypertension, can lead to the development of critical conditions in puerpera [17]. (5) Red blood cell count. To meet the needs of fetal development during pregnancy, endocrine changes occur in pregnant women, which increases the red blood cell count and improves the blood viscosity, seriously affecting the microcirculation in the body, causing ischemic and hypoxic damage to organs and increasing the risk of critical conditions [18].

Nomogram model, based on core diagnostic indicators, can be used for risky patient assessment and early disease diagnosis. It has been used effectively for various conditions, including prediction of adverse outcomes in pregnant women and fetuses with pulmonary arterial hypertension [19], radiomics-based prediction of the risk of preeclampsia in patients with hypertension during pregnancy [13], screening for new-onset hypertension in rural Chinese population [20], prediction of occult hypertension and occult uncontrolled hypertension based on clinical characteristics of single outpatient visit [21], etc. In this study, after integrating the risk factors of the multifactorial analysis results, the constructed visualized nomogram model had an AUC value of more than 0.8 in both internal and external validation, indicating good consistency and discrimination of the nomogram model herein. Clinical healthcare professionals can predict the risk of critical conditions in patients with hypertension in pregnancy based on the summation of the factors, and thus do a good job in the prevention of high-risk pregnant women with severe preeclampsia.

In conclusion, gestational hypertension patients with aged > 30 years, junior high school education or below, family history of hypertension, anemia during pregnancy, and high red blood cell count are at greater risk of developing critical conditions. Nomogram prediction model based on the above risk factors can be considered in clinical practice to reasonably assess the risk of critical conditions and targeted measures can be taken to reduce the incidence of critical conditions. However, it is important to note that this study is a retrospective study conducted at a single center and is limited by its small sample size. Therefore, further validation is necessary to confirm the reliability of the nomogram model and each risk factor. In the future, additional factors will be incorporated into the model analysis to enhance the accuracy and effectiveness of the model.

## Data Availability

The [DATA TYPE] data used to support the findings of this study are included within the article.
